# Acute clinical and financial outcomes of esophagectomy at safety-net hospitals in the United States

**DOI:** 10.1371/journal.pone.0285502

**Published:** 2023-05-24

**Authors:** Sara Sakowitz, Russyan Mark Mabeza, Syed Shahyan Bakhtiyar, Arjun Verma, Shayan Ebrahimian, Amulya Vadlakonda, Sha’shonda Revels, Peyman Benharash

**Affiliations:** 1 Cardiovascular Outcomes Research Laboratories (CORELAB), University of California, Los Angeles, CA, United States of America; 2 Department of Surgery, University of Colorado, Aurora, CO, United States of America; 3 Department of Surgery, University of California, Los Angeles, CA, United States of America; Yale School of Medicine: Yale University School of Medicine, UNITED STATES

## Abstract

**Background:**

While safety-net hospitals (SNH) play a critical role in the care of underserved communities, they have been associated with inferior postoperative outcomes. This study evaluated the association of hospital safety-net status with clinical and financial outcomes following esophagectomy.

**Methods:**

All adults (≥18 years) undergoing elective esophagectomy for benign and malignant gastroesophageal disease were identified in the 2010–2019 Nationwide Readmissions Database. Centers in the highest quartile for the proportion of uninsured/Medicaid patients were classified as SNH (others: non-SNH). Regression models were developed to evaluate adjusted associations between SNH status and outcomes, including in-hospital mortality, perioperative complications, and resource use. Royston-Parmar flexible parametric models were used to assess time-varying hazard of non-elective readmission over 90 days.

**Results:**

Of an estimated 51,649 esophagectomy hospitalizations, 9,024 (17.4%) were performed at SNH. While SNH patients less frequently suffered from gastroesophageal malignancies (73.2 vs 79.6%, p<0.001) compared to non-SNH, the distribution of age and comorbidities were similar. SNH was independently associated with mortality (AOR 1.24, 95% CI 1.03–1.50), intraoperative complications (AOR 1.45, 95% CI 1.20–1.74) and need for blood transfusions (AOR 1.61, 95% CI 1.35–1.93). Management at SNH was also associated with incremental increases in LOS (+1.37, 95% CI 0.64–2.10), costs (+10,400, 95% CI 6,900–14,000), and odds of 90-day non-elective readmission (AOR 1.11, 95% CI 1.00–1.23).

**Conclusions:**

Care at safety-net hospitals was associated with higher odds of in-hospital mortality, perioperative complications, and non-elective rehospitalization following elective esophagectomy. Efforts to provide sufficient resources at SNH may serve to reduce complications and overall costs for this procedure.

## Introduction

Despite advances in surgical technique and care, esophagectomy remains a high-risk operation that necessitates multidisciplinary management of comorbidities and postoperative complications. Whether performed for benign or malignant disease, large-scale outcomes of esophagectomy are suboptimal, with mortality and complication rates as high as 10% and 60%, respectively [1–4]. Several patient factors including advanced age, non-White race, and lower socioeconomic status have previously been shown to be associated with increased odds of mortality and complications following esophageal resection [5,6]. More importantly, center-level variation is also known to significantly influence outcomes of complex procedures such as esophagectomy [7–10].

By definition, safety-net hospitals (SNH) provide care to a higher proportion of under- or uninsured patients. Consequently, these patients are among the most vulnerable to variability in healthcare delivery and often have more complex medical profiles [11]. Mounting evidence suggests SNH to be associated with inferior clinical outcomes and higher expenditures across a multitude of surgical procedures [12–15]. However, reports on such associations in esophagectomy [11] rely on older, more limited cohorts and datasets. More recently, Gurien and colleagues [16] reported Ivor Lewis esophagectomy outcomes at a single academic safety-net center that were superior to national benchmarks. Given these conflicting findings, changes in esophageal disease screening and surgical indications, as well as the evolution of value-based healthcare in the US, a contemporary analysis of outcomes for esophagectomy at SNH is warranted.

Examining national data across a decade, we characterized the association of SNH with acute clinical and financial endpoints following elective esophagectomy for benign and malignant gastroesophageal conditions. We hypothesized that after risk adjustment, care at SNH would remain associated with increased mortality, perioperative complications, resource use, and non-elective readmission.

## Methods

**Fig 1:**
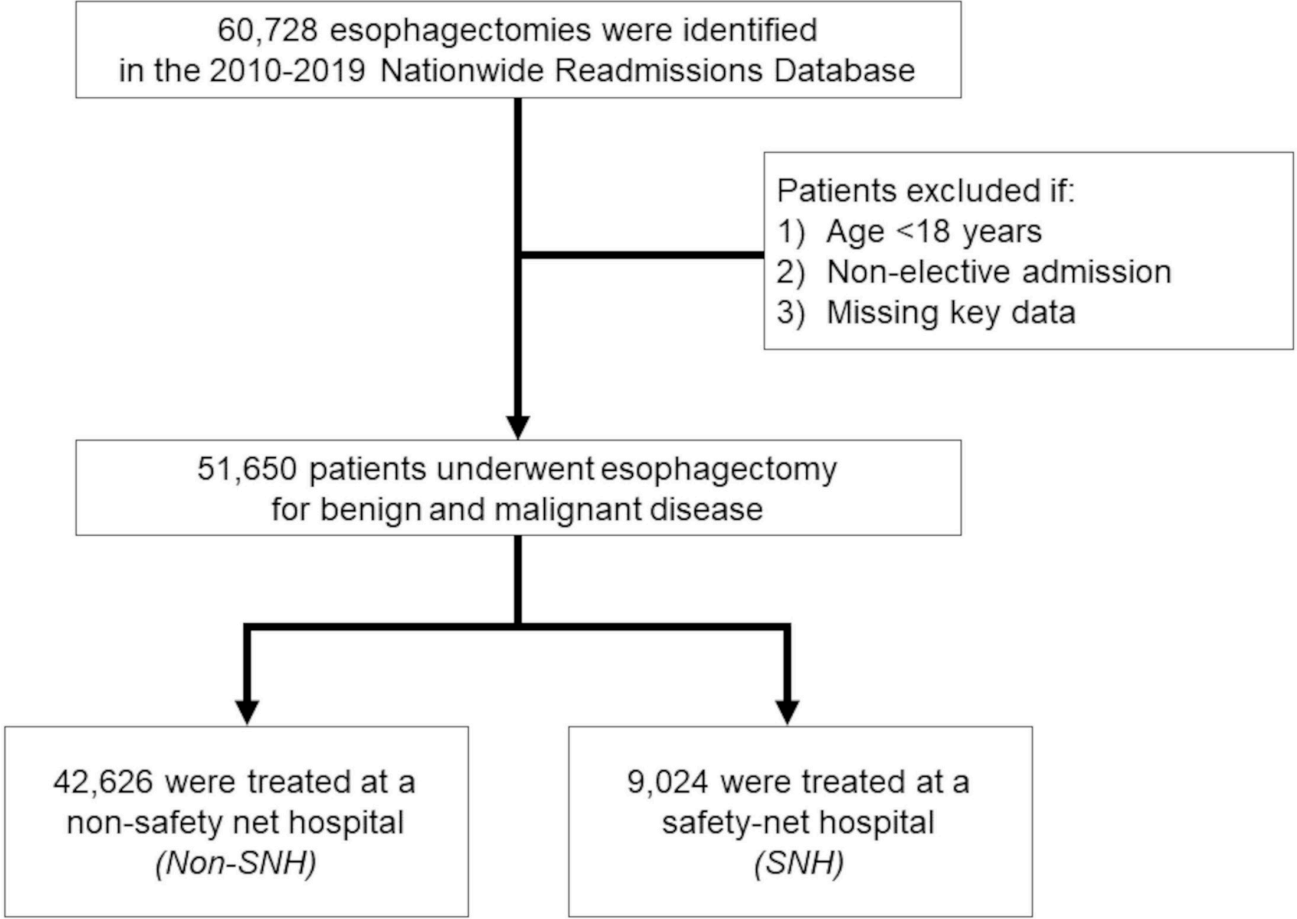
Study flowchart of survey-weighted estimates. Of 60,728 hospitalizations for elective esophagectomy identified in the 2010–2019 NRD, 51,650 patients undergoing resection for benign or malignant gastroesophageal disease were included for analysis. Of these, 9,024 (17.5%) received treatment at a safety-net hospital (SNH). All estimates represent survey-weighted methodology.

All adult (≥ 18 years) hospitalizations for elective esophagectomy with relevant diagnoses of benign or malignant gastroesophageal conditions were identified in the 2010–2019 Nationwide Readmissions Database (NRD) using previously-reported *International Classification of Diseases*, *Ninth and Tenth Revision* (ICD-9/10) diagnosis and procedure codes [17]. Maintained by the Healthcare Cost and Utilization Project (HCUP), the NRD is the largest, all-payer readmissions database that allows rehospitalizations to be tracked across hospitals within each calendar year, and accounts for nearly 60% of all U.S. hospitalizations [18]. Patients missing key data were excluded from further analysis (2.7% of patients) (Fig 1).

The HCUP data dictionary was used to define relevant patient and hospital characteristics, including age, sex, income quartile, primary payer, and hospital teaching status [19]. The Elixhauser Comorbidity Index was used to quantify patients’ burden of chronic conditions [20]. Previously described ICD-9/10 diagnosis codes were used to identify specific comorbidities and perioperative complications which were grouped into cardiac (cardiac arrest, ventricular fibrillation, ventricular tachycardia, tamponade, myocardial infarction), cerebrovascular (intracranial hemorrhage, acute ischemic complications, stroke), infectious (sepsis, surgical site infection), intraoperative (accidental puncture, hemorrhage, nerve injury), and respiratory complications (pneumonia, ARDS, respiratory failure, pneumothorax, prolonged ventilation) [21]. Operative approach was defined as open, laparoscopic, or robotic-assisted based on available ICD codes [22]. A Royston-Parmar flexible parametric model was used to ascertain the risk-adjusted cumulative hazard of readmission [23]. As the NRD does not allow patients to be tracked from year to year, we excluded patients admitted in October, November, and December of each year for 90-day readmissions analyses [18]. To estimate costs, center-specific cost-to-charge ratios were applied to overall charges, which were adjusted for inflation using the 2019 Personal Healthcare Price Index [24]. Annual esophagectomy volume was used to categorize hospitals into low-, medium-, or high-volume tertiles. Following previously-published methodology, hospitals in the top quartile of all Medicaid or self-pay admissions were considered SNH [13]. Patients treated at SNH were considered the SNH cohort (others: non-SNH).

The primary outcome of interest was in-hospital mortality, while secondary endpoints included perioperative complications, duration of index admission (LOS), hospitalization costs, and non-elective readmission within 90 days of index discharge. Categorical variables are presented as percentages (%), while continuous variables are reported as medians with interquartile range (IQR). Mann-Whitney U and Pearson’s tests were used for bivariate comparisons of continuous and categorical variables, respectively. Cuzick’s nonparametric test was used to examine the significance of temporal trends [25]. Multivariable regression models were constructed to adjust for patient and hospital differences in assessing the associations between SNH and study outcomes. Covariate selection was performed using elastic net regularization, which minimizes overfitting and collinearity via a penalized least-squares methodology [26]. Model discrimination and goodness of fit were assessed using receiver operating characteristics and the coefficient of discrimination, as appropriate.

Our primary analysis considered the impact of care at SNH on outcomes of esophagectomy for benign and malignant disease. Subsequently, we conducted three sensitivity analyses. First, we limited the cohort to those with gastroesophageal malignancy, excluding patients with benign disease. Second, we constructed a two-level (patient and hospital, respectively), mixed-effects model to assess outcomes of interest, with the random effect being the NRD hospital identifier [27,28]. Thirdly, we utilized entropy balancing to balance groups across patient, operative, and hospital factors, to isolate the relationship between SNH status and outcomes of interest. Briefly, entropy balancing applies sample weights to balance covariates, while retaining the full cohort for analysis [29–31].

Significance was considered as p≤0.05. Stata 16.1 (StataCorp, College Station, TX) was used for all statistical analyses. This study was deemed exempt from full review by the Institutional Review Board at the University of California, Los Angeles.

## Results

**Fig 2:**
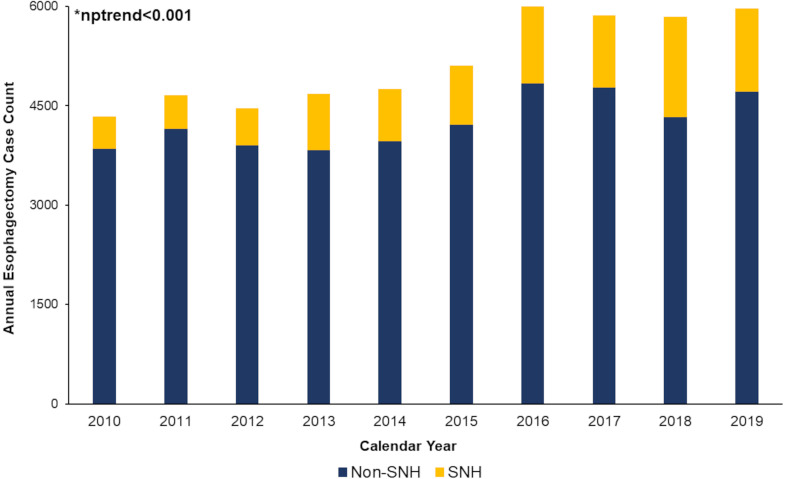
Annual trends in esophagectomy caseload and unadjusted mortality at SNH and non-SNH. Overall esophagectomy volume increased at both SNH and non-SNH (nptrend<0.001). Further, the proportion of patients undergoing esophagectomy at SNH significantly increased from 2010 to 2019, from 10.8% (469) of all operations in 2010 to 20.9% (1,246) in 2019, nptrend<0.001.

Of an estimated 51,649 elective esophagectomy hospitalizations for benign and malignant gastroesophageal disease, 9,024 (17.4%) occurred at SNH. Overall esophagectomy volume increased from 2010 to 2019, as did the proportion of patients receiving treatment at SNH (10.8% to 20.9%, nptrend<0.001) (Fig 2). SNH were less likely in the top tertile of operative volume (38.9 vs 48.6%, p<0.001), but more frequently classified as metropolitan teaching hospitals (83.0 vs 68.7%, p<0.001), compared to others (Table 1).

**Table 1:** Demographic, clinical, and hospital characteristics.

	*Non-SNH* (n = 42,626)	*SNH* (n = 9,024)	*P-value*
Age (years [IQR])	64 [57–71]	64 [56–71]	0.039
Female (%)	24.0	26.3	0.005
Elixhauser Comorbidity Index (median [IQR])	3 [2–5]	3 [2–4]	0.22
**Indication (%)**			<0.001
Benign disease	20.4	26.8	
Malignant disease	79.6	73.2	
**Approach (%)**			0.28
Open	76.9	77.0	
Laparoscopic	14.2	15.8	
Robotic	8.9	7.2	
**Income quartile (%)**			<0.001
>75%	25.6	18.5	
51–75%	27.4	24.9	
26–50%	26.8	27.9	
0–25%	20.2	28.7	
**Insurance coverage (%)**			<0.001
Private	42.7	32.4	
Medicare	48.1	46.9	
Medicaid	5.8	14.5	
Self-Payer	0.9	1.7	
Other Payer	2.4	4.5	
**Comorbidities (%)**			
Congestive heart failure	5.2	5.1	0.83
Coronary artery disease	15.0	12.9	<0.001
Peripheral vascular disease	4.2	3.0	<0.001
Pulmonary circulation disorders	2.5	2.3	0.39
Valvular heart disease	3.0	2.8	0.49
Hypertension	49.5	49.6	0.86
Cardiac arrhythmias	31.1	31.3	0.87
Chronic pulmonary disease	18.7	16.2	<0.001
Diabetes	18.0	17.9	0.95
Liver disease	4.9	5.0	0.89
Anemia	2.4	2.3	0.65
Electrolyte abnormality	28.4	33.4	<0.001
Coagulopathy	6.0	5.6	0.40
Neurological disorders	4.0	4.0	0.92
**Hospital Volume (%)**			<0.001
Lowest tertile	16.1	20.5	
Mid tertile	35.3	40.5	
Highest tertile	48.6	38.9	
**Hospital teaching status (%)**			<0.001
Non-Metropolitan	4.1	5.5	
Metropolitan Non-Teaching	27.2	11.4	
Metropolitan Teaching	68.7	83.0	

Reported as proportions unless otherwise noted. Statistical significance was set at α = 0.05.

*IQR, interquartile range.

Patients treated at SNH were more commonly female (26.3 vs 24.0%, p<0.01), but were otherwise similar in age and Elixhauser Comorbidity Index, relative to non-SNH. Moreover, the SNH cohort demonstrated a higher prevalence of coronary artery disease, peripheral vascular disease, chronic pulmonary disease, and electrolyte abnormalities (Table 1). A higher proportion of patients at SNH were classified in the lowest income quartile (28.7 vs 20.2%, p<0.001), compared to non-SNH.

Bivariate comparison of in-hospital outcomes is reported in Table 2. Although in-hospital mortality was similar among groups, patients at SNH more commonly experienced intraoperative complications (4.3 vs 3.3%, p = 0.03), non-home discharge (19.9 vs 17.3%, p<0.01), and non-elective readmission within 90-days of index discharge (18.6 vs 16.9%, p = 0.03). Furthermore, the SNH cohort experienced longer LOS and costs compared to the non-SNH cohort.

**Table 2:** Unadjusted and adjusted outcomes at safety-net hospitals (SNH) as compared to non-SNH.

	Unadjusted			Adjusted		
	*Non-SNH*	*SNH*	*P*	*SNH*	*95% CI*	*P*
**Clinical outcomes**						
In-hospital mortality	3.7	4.4	0.07	1.24	1.03–1.50	0.022
Infectious complications	11.4	12.4	0.19	1.16	1.02–1.32	0.026
Intraoperative complications	3.3	4.3	0.025	1.45	1.20–1.74	<0.001
Respiratory complications	27.1	26.9	0.87	1.12	1.01–1.24	0.035
Blood transfusion	13.0	15.1	0.18	1.61	1.35–1.93	<0.001
Any complication	37.6	39.3	0.20	1.15	1.05–1.26	0.004
Failure to rescue	10.7	12.6	0.09	1.22	0.99–1.49	0.06
Non-home discharge	17.3	19.9	0.004	1.21	1.08–1.36	0.002
Non-elective 90-day readmission	16.9	18.6	0.030	1.12	1.02–1.23	0.015
**Resource utilization**						
Length of stay (days) [IQR]	9 [7–15]	10 [7–16]	<0.001	+1.37	0.64–2.10	<0.001
Cost (USD $1,000) [IQR]	39 [28–61]	47 [33–69]	<0.001	+10.42	6.88–13.96	<0.001

Outcomes reported as proportions or as Adjusted Odds Ratio (AOR) with 95% confidence intervals (95% CI).

*IQR, interquartile range; *USD*, United States dollar.

**Fig 3:**
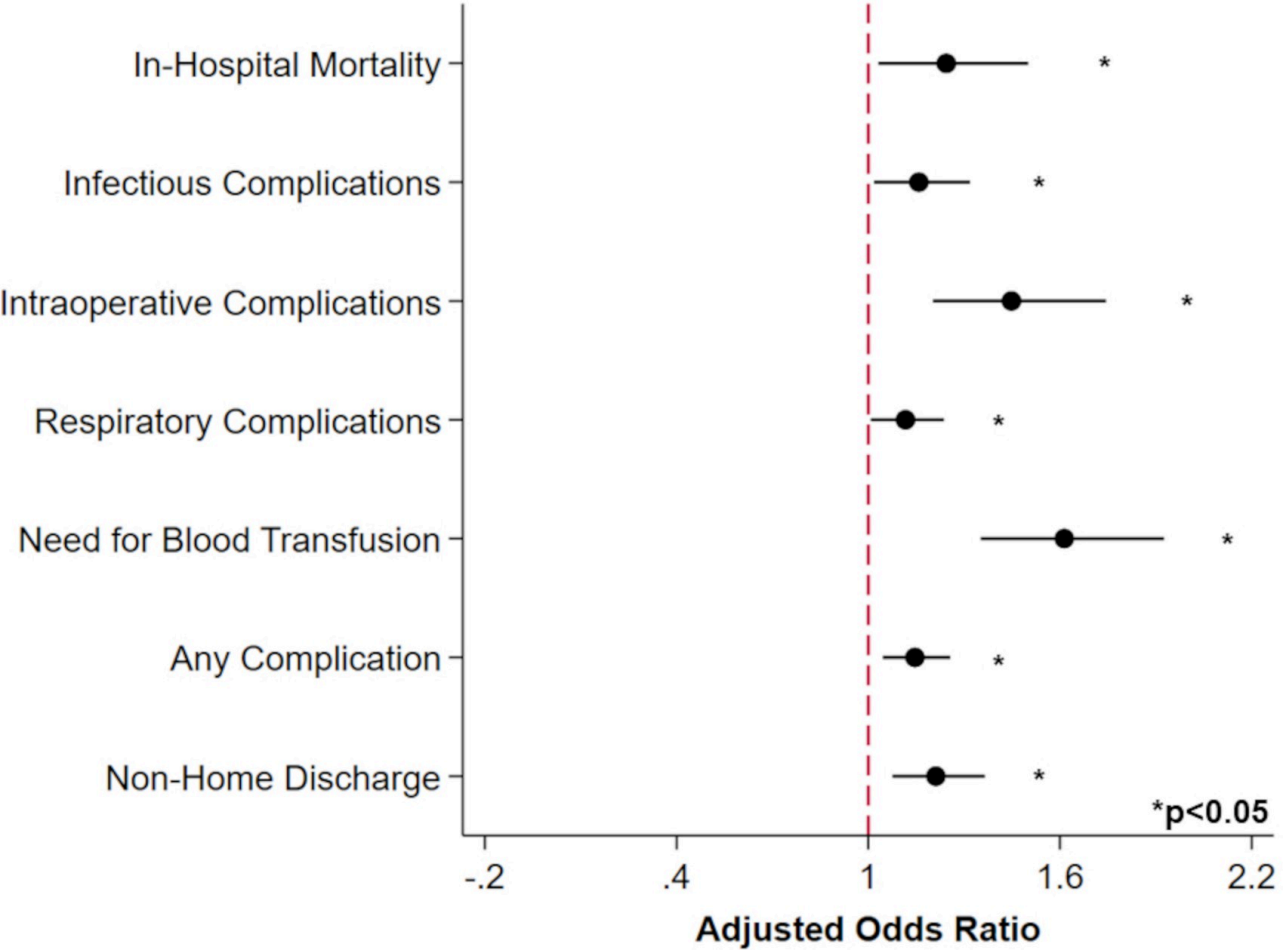
Association of hospital safety-net status with select outcomes of interest. After risk adjustment, patients undergoing esophagectomy at a safety-net hospital demonstrated greater odds of in-hospital mortality, perioperative complications, and non-home discharge. Reference: Non-safety net hospitals. Error bars represent 95% confidence intervals.

**Fig 4:**
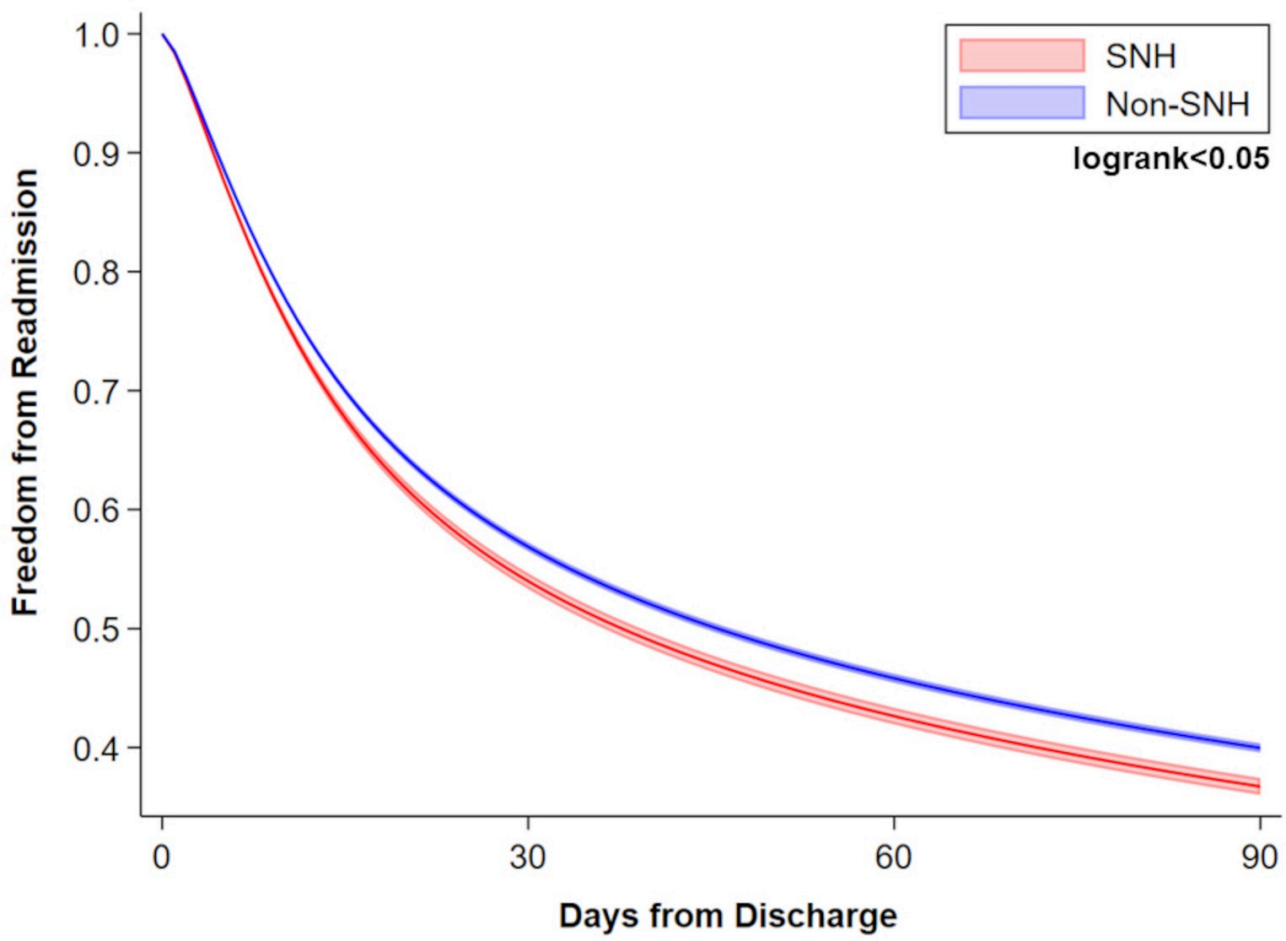
SNH is associated with greater non-elective readmission within 90 days of index discharge. A Royston-Parmar flexible parametric model was used to derive the risk-adjusted cumulative risk of readmission within 90 days of index discharge. SNH (red line) was independently associated with greater odds of readmission (AOR 1.11; 95%CI 1.00–1.23), as compared to non-SNH (blue line).

Following risk adjustment, treatment at SNH was associated with several adverse outcomes (Fig 3). Notably, SNH was linked to greater odds of in-hospital mortality (AOR 1.24, 95%CI 1.03–1.50), relative to non-SNH. Safety-net status was also associated with increased infectious (AOR 1.16, 95%CI 1.02–1.32), intraoperative (AOR 1.45, 95%CI 1.20–1.74), and respiratory complications (AOR 1.12, 95%CI 1.01–1.24), as well as need for blood transfusion (AOR 1.61, 95%CI 1.35–1.93). SNH was further associated with a 21% increase in relative odds of non-home discharge (95% CI 1.08–1.36), a 1.37-day incremental increase in hospital LOS (95% CI 0.64–2.10, p<0.001), and an additional $10,420 in costs per patient (95% CI $6,880-$13,960). Lastly, SNH was found to be linked to greater risk of readmission (AOR 1.11; 95%CI 1.00–1.23), as demonstrated using a Royston-Parmar parametric model (Fig 4).

A sensitivity analysis was conducted to evaluate independent associations between SNH and outcomes following esophagectomy for gastroesophageal malignancy, thereby excluding benign disease. Upon unadjusted comparison, patients at SNH were less likely to be treated at a high-volume center, but more likely to receive care at a metropolitan teaching hospital (S1 Table). Multivariable regression demonstrated similar associations between SNH status and in-hospital mortality, intraoperative complications, and requirement for blood transfusion. Similarly, SNH was linked to incremental increases in LOS and hospitalization costs, with non-SNH as reference. (S2 Table). A second sensitivity analysis was conducted using multi-level, mixed effects modeling. Following risk adjustment, we noted similar findings as in our primary analysis, such that SNH was linked with inferior mortality, complications, and resource utilization (S3 Table). Lastly, a third sensitivity analysis was performed following the application of entropy balancing. As in our previous analyses, SNH remained associated with greater odds of mortality, complications, and non-elective readmission, as well as increased length of stay and hospitalization expenditures (S4 Table).

## Discussion

Given the high-risk and resource-intensive nature of esophagectomy, we sought to characterize clinical and financial outcomes at safety-net hospitals using the largest readmissions database in the United States. Most commonly large, metropolitan teaching institutions, SNH were found to serve a majority lower-income population, as previously reported [22–32]. We identified a significant increase in the annual proportion of procedures performed at SNH. Safety-net status was associated with greater in-hospital mortality, adverse events, and readmission. Additionally, safety-net burden was associated with increased LOS and hospitalization costs. These findings highlight considerable resource-driven disparities in outcomes that merit further discussion.

A growing body of literature has reported adverse surgical outcomes at SNH [11–15]. Building on these studies, we found SNH to be associated with greater in-hospital complications across various domains. First, patients undergoing esophagectomy in SNH experienced a 24% increase in adjusted odds of in-hospital mortality. In addition, SNH patients experienced higher rates of intraoperative, bleeding, infectious, and respiratory complications. Notably, patients treated at SNH did not present with a significantly higher comorbidity burden, as measured via Elixhauser index, and were similarly likely to receive minimally invasive surgery, relative to non-SNH. However, patients at SNH have been reported to more frequently present with advanced disease stage [33], which is uncaptured in this dataset. Yet, considering our predictive models adjust for a comprehensive array of patient, procedural, and hospital factors, our investigation suggests these inferior outcomes are linked to intrinsic aspects of SNH. Some have proposed these sequelae may be due to delays or inefficiencies in care or insufficient implementation of quality-control protocols, all engendering substandard surgical performance [34–36]. Although contributing factors may be hospital- or region-specific, further investigation to further elucidate sources of these disparities is needed to guide systemic efforts to achieve health equity.

In the present study, we found safety-net status to be linked to a >1 day increase in hospitalization duration and an over $10,000 increase in attributable hospitalization costs. Such an increase in resource use may stem from higher rates of surgical complications [11], but may also be related to the larger financial burden incurred during the care of patients with greater comorbidity burdens [37] and more complex social needs [36]. SNH rely on low operating margins to start, so these greater patient expenditures may further limit the resources that could be devoted to quality improvement efforts [38,39]. Many SNH receive Medicaid Disproportionate Share Hospital (DSH) payments, which are meant to address the greater resource requirements experienced by centers with disproportionately high numbers of low-income patients [40]. However, the ACA reduced DSH “bridge” funding, with the expectation that more uninsured patients would be covered by Medicaid and private insurance [41]. Our results highlight the importance of continued advocacy to ensure adequate resource allocation for SNH. Yet, we recognize that changes in policy to enhance resources at SNH may take many years. In the interim, studies should identify and share institution-specific protocols that most significantly contribute to improved postoperative outcomes at high-performing centers. Whether due to enhanced recovery protocols, standardized care pathways, or post-discharge follow-up, some of these strategies may be possible to implement even in low-resource settings and could provide a financial benefit by reducing hospitalization costs and readmission rates. Ultimately, however, systemic reforms are required to address the continued inequities in clinical and financial outcomes at SNH.

Additionally, inferior outcomes at SNH may be indicative of the consequences of resource deficiency not only in the intraoperative setting, but also in outpatient medical management after these procedures. In concordance with previous studies that reported higher rates of readmission among SNH patients, this investigation found SNH to be linked to an 11% increase in adjusted odds of 30-day readmission [37]. Socioeconomically disadvantaged patients may lack access to longitudinal primary care or social support to ensure adequate recovery [42]. Compounding structural factors, including hospital distance and lack of transportation, may pose significant additional obstacles preventing low-income patients from accessing adequate follow-up [43,44]. With these significant financial disparities and increased opportunity costs, continued focus should be directed towards addressing relative resource scarcity in order to improve outcomes at SNH. Indeed, patients treated at SNH may face a variety of systemic social, environmental, residential, or economic barriers that affect health. As previous work has linked increased social services with improved patient outcomes [45,46], future interventions should consider the impact of strengthening and expanding support for families, housing, employment, or older adults. Further, given penalty disbursement has been found to regressively affect SNH, relative to non-SNH [38,39], some have suggested the Centers for Medicare and Medicaid redesign reimbursement penalty programs to adjust for social risk [47]. Systemic reforms are required at the patient, hospital, and regional level to address persistent disparities and improve care for these vulnerable patients.

Altogether, our work aligns with extensive prior literature that has identified esophagectomy as a high-risk operation across hospital safety net burden. Using the NRD we identified unadjusted in-hospital mortality rates of 3.7% and 4.4% at SNH and non-SNH, respectively. These rates accord with previously-described operative mortality rates determined using the Society of Thoracic Surgeons (STS) registry, SEER Medicare, American College of Surgeons National Surgical Quality Improvement (NSQIP) database, and the Japanese National Clinical Database [11,48–52]. However, both the STS and NSQIP registries require elective participation and are noted to capture just a small proportion of esophagectomies performed each year. In contrast, the NRD and related administrative databases maintained by HCUP draw data directly from 31 State Inpatient Databases and allow for broader and more accurate analyses of nationwide trends [18,53]. Thus, we elected to use the NRD for our national analysis of esophagectomy outcomes at safety-net hospitals over the last decade. Yet, we believe our work complements prior literature that has utilized other data for risk assessment. Whereas the NRD may allow for comprehensive national analyses of risk and readmission across hospital types, the STS database adds relevant clinical datapoints such as anastomotic leak rates and forced expiratory volume measurements [54]. Both of these perspectives are ultimately important to improve outcomes following esophagectomy for all patients.

This study has several important limitations. The NRD is an administrative database that is subject to different coding practices across participating institutions. Certain granular information, such as laboratory values, stage or extent of disease, radiographic data, and surgical margins, was unavailable for analysis. We were additionally unable to determine the use of neoadjuvant chemotherapy or radiation in the study population. Further, there were certain social factors that could impact patient care but were not possible to include for risk-adjustment, including language barriers, health literacy, or environmental factors. We also could not identify timing of diagnosis. Indeed, patients at SNH may have experienced delays from diagnosis to resection, which would undoubtedly affect their post-surgical outcomes [55,56]. Although we could not access information regarding surgeon or center experience, we controlled for annual hospital esophagectomy volume in our analysis. While the NRD provides hospitalization charges and costs, we cannot ascertain the ultimate patient’s cost burden nor the degree of hospital economic compromise. Additionally, hospital-level data, including information on care pathways, adherence to quality metrics, or timing of care, was not available. Finally, the NRD does not allow for the calculation of the STS PROM score nor other risk scores that could be used for validation. However, we found a similar C-statistic for our regression model (0.75) as what has been previously published for STS PROM [57]. Despite these limitations, we used robust statistical methodology and the largest all-payer dataset to report on contemporary and nationally representative outcomes following esophagectomy.

In conclusion, an increasing proportion of esophagectomy patients is receiving care at safety-net hospitals across the US. Operations at SNH appear associated with increased mortality, perioperative complications, longer inpatient stays and higher costs. Furthermore, SNH patients experienced higher rates of readmission relative to their non-SNH counterparts. Given the essential role SNH play in the care of vulnerable populations, novel policies are needed to protect the economic viability of these centers and improve quality and efficiency of care. Additionally, healthcare reform must confront the fragmented system of outpatient care that many of these patients face. By revealing persistent inequities in postoperative outcomes, we hope to promote conversation, novel interventions, and policy change to address these issues at local, regional, and national levels. Ultimately, both safety-net and non-safety net hospitals should strive to address continued disparities and promote equitable outcomes in esophagectomy.

## Supporting information

S1 Table Demographic, clinical, and hospital characteristics for patients undergoing esophagectomy for gastroesophageal malignancies. Reported as proportions unless otherwise noted. Statistical significance was set at α = 0.05. *IQR, interquartile range, *SMD, standardized mean difference.
(DOCX)

S2 Table Unadjusted and adjusted outcomes of patients undergoing esophagectomy for gastroesophageal malignancies at safety-net hospitals (SNH) as compared to non-SNH. Outcomes reported as proportions or as Adjusted Odds Ratio (AOR) with 95% confidence intervals (95% CI). *IQR, interquartile range; *USD*, United States dollar.
(DOCX)

S3 Table Adjusted outcomes of patients undergoing esophagectomy at safety-net hospitals (SNH) as compared to non-SNH, following multi-level, mixed effects modeling with random effect being NRD hospital identifier. CAPTION: Outcomes reported as Adjusted Odds Ratio (AOR) with 95% confidence intervals (95% CI). *IQR, interquartile range; *USD*, United States dollar.
(DOCX)

S4 Table Adjusted outcomes of patients undergoing esophagectomy at safety-net hospitals (SNH) as compared to non-SNH, following entropy balancing. CAPTION: Outcomes reported as Adjusted Odds Ratio (AOR) with 95% confidence intervals (95% CI). *IQR, interquartile range; *USD*, United States dollar.
(DOCX)
