# Supplementary material for: Acute clinical and financial outcomes of esophagectomy at safety-net hospitals in the United States
Source: PLoS One. 2023 May 24;18(5):e0285502. doi: 10.1371/journal.pone.0285502 (PMC10208475; doi:10.1371/journal.pone.0285502)
Supplement: S1 Table — Reported as proportions unless otherwise noted. Statistical significance was set at α = 0.05. *IQR, interquartile range, *SMD, standardized mean difference. (DOCX) [file pone.0285502.s001.docx]

**S1 Table:**

TITLE: Demographic, clinical, and hospital characteristics for patients undergoing esophagectomy for gastroesophageal malignancies.

CAPTION: Reported as proportions unless otherwise noted. Statistical significance was set at α= 0.05.

**IQR*, interquartile range*, *SMD*, standardized mean difference

|  | ***Non-SNH***  (n = 33,916) | ***SNH***  (n = 6,603) | ***P-value*** | ***SMD*** |
| --- | --- | --- | --- | --- |
| Age (years [IQR]) | 64 [58-71] | 65 [58-71] | 0.61 |  |
| Female (%) | 17.6 | 18.8 | 0.17 | 0.02 |
| Elixhauser Comorbidity Index (median [IQR]) | 4 [3-5] | 4 [3-5] | 0.66 |  |
| **Approach (%)** |  |  | 0.43 | 0.04 |
| Open | 76.6 | 77.8 |  |  |
| Laparoscopic | 14.2 | 14.9 |  |  |
| Robotic | 9.2 | 7.4 |  |  |
| **Income quartile (%)** |  |  | <0.001 | 0.26 |
| >75% | 26.4 | 18.6 |  |  |
| 51-75% | 27.5 | 25.6 |  |  |
| 26-50% | 26.6 | 28.3 |  |  |
| 0-25% | 19.5 | 27.5 |  |  |
| **Insurance coverage (%)** |  |  | <0.001 | 0.31 |
| Private | 43.8 | 32.5 |  |  |
| Medicare | 47.7 | 48.7 |  |  |
| Medicaid | 5.4 | 13.0 |  |  |
| Self-Payer | 0.7 | 1.4 |  |  |
| Other Payer | 2.3 | 4.3 |  |  |
| **Comorbidities (%)** |  |  |  |  |
| Congestive heart failure | 5.3 | 5.4 | 0.80 | 0.01 |
| Coronary artery disease | 15.8 | 14.3 | 0.037 | 0.02 |
| Peripheral vascular disease | 4.6 | 3.2 | <0.001 | 0.08 |
| Pulmonary circulation disorders | 2.8 | 2.2 | 0.08 | 0.03 |
| Valvular heart disease | 2.9 | 2.8 | 0.81 | 0.02 |
| Hypertension | 49.9 | 51.3 | 0.24 | 0.03 |
| Cardiac arrhythmias | 34.4 | 36.8 | 0.037 | 0.03 |
| Chronic pulmonary disease | 18.6 | 15.9 | 0.002 | 0.06 |
| Diabetes | 18.9 | 18.5 | 0.57 | 0.01 |
| Liver disease | 5.3 | 5.6 | 0.52 | 0.01 |
| Anemia | 2.6 | 2.5 | 0.77 | 0.02 |
| Electrolyte abnormality | 30.7 | 36.1 | <0.001 | 0.12 |
| Coagulopathy | 6.7 | 6.4 | 0.68 | 0.02 |
| Neurological disorders | 3.9 | 3.8 | 0.78 | 0.01 |
| **Hospital Volume (%)** |  |  | 0.013 | 0.05 |
| Lowest tertile | 1.8 | 3.1 |  |  |
| Mid tertile | 9.0 | 10.5 |  |  |
| Highest tertile | 89.1 | 86.5 |  |  |
| **Hospital teaching status (%)** |  |  | <0.001 | 0.30 |
| Non-Metropolitan | 1.0 | 0.6 |  |  |
| Metropolitan Non-Teaching | 9.1 | 2.5 |  |  |
| Metropolitan Teaching | 89.9 | 96.9 |  |  |
