# Supplementary material for: Acute clinical and financial outcomes of esophagectomy at safety-net hospitals in the United States
Source: PLoS One. 2023 May 24;18(5):e0285502. doi: 10.1371/journal.pone.0285502 (PMC10208475; doi:10.1371/journal.pone.0285502)
Supplement: S2 Table — Outcomes reported as proportions or as Adjusted Odds Ratio (AOR) with 95% confidence intervals (95% CI). *IQR, interquartile range; USD, United States dollar. (DOCX) [file pone.0285502.s002.docx]

**S2 Table**:

TITLE: Unadjusted and adjusted outcomes of patients undergoing esophagectomy for gastroesophageal malignancies at safety-net hospitals (SNH) as compared to non-SNH.

CAPTION: Outcomes reported as proportions or as Adjusted Odds Ratio (AOR) with 95% confidence intervals (95% CI).

**IQR*, interquartile range; *USD,* United States dollar

|  | **Unadjusted*** | | | **Adjusted^†^** | | |
| --- | --- | --- | --- | --- | --- | --- |
|  | ***Non-SNH*** | ***SNH*** | ***P*** | ***SNH*** | ***95% CI*** | ***P*** |
| **Clinical outcomes** |  |  |  |  |  |  |
| In-hospital mortality | 3.7 | 4.4 | 0.07 | 1.25 | 1.01 - 1.55 | 0.036 |
| Infectious complications | 11.4 | 12.4 | 0.19 | 1.08 | 0.95 - 1.25 | 0.25 |
| Intraoperative complications | 3.3 | 4.3 | 0.026 | 1.50 | 1.20 - 1.87 | <0.001 |
| Respiratory complications | 27.1 | 26.9 | 0.87 | 1.07 | 0.95 - 1.20 | 0.25 |
| Blood transfusion | 13.0 | 15.1 | 0.18 | 1.77 | 1.46 - 2.15 | <0.001 |
| Cerebrovascular complications | 0.4 | 0.5 | 0.74 | 1.18 | 0.56 - 2.47 | 0.67 |
| Thromboembolic complications | 2.6 | 2.3 | 0.37 | 1.18 | 0.85 - 1.63 | 0.32 |
| Any complication | 37.6 | 39.3 | 0.20 | 1.12 | 1.00 - 1.24 | 0.041 |
| Failure to rescue | 10.7 | 12.6 | 0.09 | 1.23 | 0.98 - 1.54 | 0.08 |
| Non-home discharge | 17.3 | 19.9 | 0.004 | 1.24 | 1.09 - 1.40 | 0.001 |
| Non-elective 90-day readmission | 16.9 | 18.6 | 0.030 | 1.11 | 1.00 - 1.23 | 0.045 |
| **Resource utilization** |  |  |  |  |  |  |
| Length of stay (days) [IQR] | 10 [8-15] | 11 [8-16] | <0.001 | +1.12 | 0.40 - 1.84 | 0.002 |
| Cost (USD $1,000) [IQR] | 42 [31-63] | 50 [37-72] | <0.001 | +9.33 | 5.88 - 12.77 | <0.001 |
