# Supplementary material for: Acute clinical and financial outcomes of esophagectomy at safety-net hospitals in the United States
Source: PLoS One. 2023 May 24;18(5):e0285502. doi: 10.1371/journal.pone.0285502 (PMC10208475; doi:10.1371/journal.pone.0285502)
Supplement: S3 Table — CAPTION: Outcomes reported as Adjusted Odds Ratio (AOR) with 95% confidence intervals (95% CI). *IQR, interquartile range; USD, United States dollar. (DOCX) [file pone.0285502.s003.docx]

**S3 Table:**

TITLE: Adjusted outcomes of patients undergoing esophagectomy at safety-net hospitals (SNH) as compared to non-SNH, following multi-level, mixed effects modeling with random effect being NRD hospital identifier.

CAPTION: Outcomes reported as Adjusted Odds Ratio (AOR) with 95% confidence intervals (95% CI).

**IQR*, interquartile range; *USD,* United States dollar

|  | **Adjusted Multivariable Regression** | |
| --- | --- | --- |
|  | **SNH** | **95%CI** |
| *Clinical outcomes [AOR]* |  |  |
| In-hospital mortality | **1.25** | **1.02-1.52** |
| Infectious complications | **1.18** | **1.05-1.33** |
| Intraoperative complications | **1.62** | **1.24-2.11** |
| Respiratory complications | **1.27** | **1.11-1.46** |
| Blood transfusion | **2.15** | **1.71-2.71** |
| Cerebrovascular complications | 1.17 | 0.49-2.84 |
| Any complication | **1.24** | **1.10-1.40** |
| Non-home discharge | **1.22** | **1.04-1.42** |
| Non-elective 30 day readmission | 1.14 | 0.99-1.30 |
| *Resource utilization* |  |  |
| Length of stay (days) [β] | **+1.59** | **+0.84-2.34** |
| Cost (USD $1,000) [β] | **+11.28** | **+7.07-15.48** |
