# Supplementary material for: Acute clinical and financial outcomes of esophagectomy at safety-net hospitals in the United States
Source: PLoS One. 2023 May 24;18(5):e0285502. doi: 10.1371/journal.pone.0285502 (PMC10208475; doi:10.1371/journal.pone.0285502)
Supplement: S4 Table — CAPTION: Outcomes reported as Adjusted Odds Ratio (AOR) with 95% confidence intervals (95% CI). *IQR, interquartile range; USD, United States dollar. (DOCX) [file pone.0285502.s004.docx]

**S4 Table:**

TITLE: Adjusted outcomes of patients undergoing esophagectomy at safety-net hospitals (SNH) as compared to non-SNH, following entropy balancing.

CAPTION: Outcomes reported as Adjusted Odds Ratio (AOR) with 95% confidence intervals (95% CI).

**IQR*, interquartile range; *USD,* United States dollar

|  | **Adjusted** | | |
| --- | --- | --- | --- |
|  | ***SNH*** | ***95% CI*** | ***P*** |
| **Clinical outcomes** |  |  |  |
| In-hospital mortality | 1.32 | 1.09-1.59 | 0.01 |
| Infectious complications | 1.18 | 1.03-1.34 | 0.01 |
| Intraoperative complications | 1.37 | 1.14-1.66 | 0.001 |
| Respiratory complications | 1.14 | 1.03-1.27 | 0.01 |
| Blood transfusion | 1.42 | 1.17-1.73 | <0.001 |
| Cerebrovascular complications | 1.48 | 0.81-2.70 | 0.20 |
| Thromboembolic complications | 1.17 | 0.85-1.61 | 0.33 |
| Any complication | 1.18 | 1.07-1.30 | 0.001 |
| Failure to rescue | 1.28 | 1.05-1.56 | 0.02 |
| Non-home discharge | 1.22 | 1.08-1.37 | 0.001 |
| Non-elective 90-day readmission | 1.15 | 1.04-1.26 | 0.004 |
| **Resource utilization** |  |  |  |
| Length of stay (days) [IQR] | +1.43 | +0.71-2.15 | <0.001 |
| Cost (USD $1,000) [IQR] | +10.90 | +7.41-14.39 | <0.001 |
